# New guidance to improve sample size calculations for trials: eliciting the target difference

**DOI:** 10.1186/s13063-018-2894-y

**Published:** 2018-11-05

**Authors:** Melanie L. Bell

**Affiliations:** Department of Epidemiology and Biostatistics, Mel and Enid Zuckerman College of Public Health, University of Arizona, Tucson, AZ 85724 USA

**Keywords:** Effect size, Target difference, Power, Sample size, Randomized controlled trials

## Abstract

**Background:**

Sample size calculations are central to the design of health research trials. To ensure that the trial provides good evidence to answer the trial’s research question, the target effect size (difference in means or proportions, odds ratio, relative risk or hazard ratio between trial arms) must be specified under the conventional approach to determining the sample size. However, until now, there has not been comprehensive guidance on how to specify this effect.

**Main text:**

This is a commentary on a collection of papers from two important projects, DELTA (Difference ELicitation in TriAls) and DELTA^2^ that aim to provide evidence-based guidance on systematically determining the target effect size, or difference and the resultant sample sizes for trials. In addition to surveying methods that researchers are using in practice, the research team met with various experts (statisticians, methodologists, clinicians and funders); reviewed guidelines from funding agencies; and reviewed recent methodological literature. The DELTA^2^ guidance stresses specifying important and realistic differences, and undertaking sensitivity analyses in calculating sample sizes. It gives recommendations on how to find appropriate differences, conduct the sample size calculation(s) and how to report these in grant applications, protocols and manuscripts. It is hoped that this will contribute not only to better powered studies, but better reporting and reproducibility and thinking about what a trial should be designed to achieve.

**Conclusions:**

The DELTA researchers have developed a set of comprehensive guidance documents that are welcome and will almost certainly improve the way that trials are designed and reported.

## Background

Most applied statisticians working in health research have experienced a form of the following interchange when discussing trial design with an investigator.

Biostatistician: What is the difference that we should base our sample size calculations on?

Investigator: I do not know, what is it?

Biostatistician: Uh…you are supposed to tell me.

Investigator: But…how would I know?

What follows is likely to be a muddled conversation about important differences (what is important?), plausibility (how do we determine that?) and budget (we cannot afford any more than *n* = *x*).

Despite the paramount importance of an a-priori sample size calculation, until now there has not been comprehensive guidance in specifying the target effect size, or difference. It can be expressed as the difference in means or proportions, odds ratio, relative risk or hazard ratio between arms. The target difference is a key quantity in sample size calculation, and is the most difficult to determine, as most other quantities are fixed (e.g., type I error rate = 0.05, power = 80 or 90%) or are parameters that can be estimated (standard deviation, control group event proportion). The sample size required is highly sensitive to the chosen target difference: a halving of the difference in means results in quadruple the required sample size for a balanced two-arm randomized controlled trial (RCT), for example. Thus, a carefully considered target difference is key. A strong case can also be made that not only does improper powering have resource implications, there are ethical issues as well: underpowering a study to detect important differences can expose patients to the risks and burden of research with little chance for a definitive answer. On the other hand, overpowered studies may find statistically significant, but not clinically important differences, and may expose more participants to research risks than necessary to answer a clinically relevant research question [1]. This is a commentary on a collection of five papers on sample size calculations and specifying the target difference for randomized trials, based on two studies described below [2–6]. Included in these papers is an upcoming Medical Research Council guidance [2]. A full Health Technology Assessment report based upon the first project is also available [7].

## Main text

Cook et al. have carried out projects called DELTA (Difference ELicitation in TriAls) and DELTA^2^ that aim to provide evidence-based guidance on systematically determining the target difference and the resultant sample sizes in trials. The original DELTA study undertook a systematic review to determine the methods that researchers are using in practice [7] and carried out two surveys among trialists. Based on these, an initial guidance document was developed [3]. The DELTA^2^ study extended the scope of the original project to better meet the needs of researchers and funders for estimation and reporting of the target difference. The research team reviewed funding agency guidelines and recent methodological literature, and updated the original DELTA guidelines to include new approaches for specifying a target difference. They gathered input from experts and stakeholders (statisticians, methodologists, clinicians and funders) through a Delphi study, and engagement sessions [6]. The results of these two projects are comprehensive guidance documents that are welcome and will almost certainly improve the way that trials are designed and reported [2, 3].

The two reviews found that seven methods are in use for specifying differences in sample size calculations: anchor, distribution, health economics, opinion-seeking, pilot studies, review of evidence, and standardized effect sizes. The Rothwell et al. 2018 review found the most common approach was the review of evidence (46%) [5]; the Cook et al. 2014 review found that the anchor method was the most common approach (33%), with many studies using multiple methods [7]. The difference between reviews may be that the latter review only included manuscripts in a single journal, possibly reflecting a particular subtype of trials. See the new guidance, in this issue, for more detail on each of the methods [2].

The full DELTA^2^ guidance document contains detailed background and examples, which will help to ensure translation into practice. Information on the seven methods from above are outlined and well-referenced. The appendix outlines conventional approaches to RCT sample size calculation; alternative approaches including precision of estimation; Bayesian methods and value of information; and alternative trial designs including the increasingly popular adaptive designs. Several reporting exemplars are given. The result is a rich information source that even includes a summary in lay language for patient and public contributors.

The DELTA^2^ guidance largely focuses on important and realistic/plausible differences and gives detailed information on how to assess these qualities. Key recommendations are to search the literature to inform the target difference; explore candidate primary outcomes; ensure that the views of stakeholders are considered so that importance can be addressed; investigate and justify importance and plausibility (i.e., a realistic difference); use existing studies to estimate other parameters (standard deviation, baseline hazard, control group proportion); and perform sensitivity analyses.

The DELTA^2^ group stressed importance in their guidance for specifying the target difference. As the beginning vignette suggests, sample size calculations can be confusing, and the issue of importance may be the slipperiest factor. What is a meaningful effect, and how does one determine what it is? Would it make a difference to patients? Change clinical practice? Affect policy? Answers to these questions can have a subjective aspect to them, which may make some researchers uncomfortable. However, researchers in developing and evaluating patient-reported outcomes have grappled with the concept of meaningful effects, and recommend the anchor method, where some external assessment (clinical or patient based) is used to classify subjects based on their levels of change [8]. The aforementioned reviews found that many researchers rely on pilot data or a review of the evidence base to determine the target effect size [5, 7]. Pilot data can address plausibility (is it possible to actually find this difference?), but not importance, and may mislead researchers in their trial design [9]. Review of the evidence may be similar—a statistically significant difference that some other research group(s) found may not indicate importance, so care must be taken to also ascertain relevance to stakeholders (patients, clinicians, policy-makers (opinion-seeking)). Despite the DELTA^2^ guidance, I believe that many basic/bench scientists will still struggle with specifying important differences for their experiments’ sample sizes. Difference between arms in a weight loss trial, for example, will almost certainly be easier to assess for importance as compared to many basic science outcomes. However, the guidelines should help those scientists to begin to think about designing their experiments to detect important differences.

The attention to sensitivity analyses is welcome. Sensitivity analyses are increasingly being recognized as important for assessing the robustness of results to assumptions of the primary analysis [10], but perhaps have not been used as much during trial design. Sensitivity analyses can be undertaken at the trial planning stage by varying key inputs to the sample size calculations [11].

The DELTA^2^ guidance gives detailed guidance on reporting for grant applications, protocols and manuscripts, and thus may contribute not only to better-powered studies, but better reporting and reproducibility. With the introduction of the CONSORT (CONsolidated Standards Of Reporting Trials) Statement in 1993, the importance of reporting trial methods and results in a consistent and transparent fashion has been increasingly recognized, and there are indications that reporting has improved [12]. Reporting of sample size calculations has also increased: 4% in 1980 (high- and low-impact journals), 83% in 2002 (five leading medical journals) and 95% in 2006 (six high-impact factor journals) [13], although the sampling frame is also likely to be associated with these differences. Despite problems with replicability of these calculations, either due to missing information [13], or possibly due to differences in software [14], the increasing trend indicates a recognition of the importance of statistical power and the sample size calculation.

The DELTA^2^ guidance recommendations discuss financial considerations for the trial only with respect to a certain type of health economic method, the “value of information” approach. This approach, which factors in trial cost and cost per unit increase in health, is rarely used in practice. However, funding priorities for granting agencies and their budget constraints usually play a substantial part in the design of a trial. If the required sample size is too large because of trial budgetary considerations, researchers may (1) implement procedures in the conduct of the study to reduce missing data and dropout [15, 16]; (2) choose a primary outcome that is more responsive [17]; or (3) use a surrogate or intermediate outcome that is cheaper to assess or will have more events. Missing data reduce sample size (as well as inducing bias, in many cases) and although missing data are inevitable, there are ways to minimize it [15, 16]. Further work should be undertaken to incorporate budget considerations into sample size calculations.

## Conclusion

Stephen Senn once quipped that sample size estimation is a guess masquerading as mathematics [18]. The DELTA^2^ guidance will help to reduce the guesswork, and this should translate into better health research.
